# ARHGEF2 Isoform Switching Couples Intestinal Epithelial Barrier Function with Autophagic Response to Pathogens

**DOI:** 10.21203/rs.3.rs-8252764/v1

**Published:** 2025-12-11

**Authors:** Shuyuan Chen, Alka Diwaker, Xi Li, Abdulmajeed Salamah, Lei Guo, Neethu S. Alex, Ashutosh Shukla, Mary-Joe Touma, Yassin EL-Najjar, Lin Xu, Jianyi Yin, Ezra Burstein, Marcel Mettlen, Andrew Lemoff, Suraj Patel, Josephine Ni, Hans-Christian Reinecker

**Affiliations:** 1 Department of Medicine, Division of Digestive and Liver Diseases, University of Texas Southwestern Medical Center, 5959 Harry Hines Boulevard, Dallas, Texas 75390, United States; 2 Quantitative Biomedical Research Center, Peter O’Donnell Jr. School of Public Health, University of Texas Southwestern Medical Center, Texas 75390, United States; 3 Department of Cell Biology, University of Texas Southwestern Medical Center, Dallas, TX 75390, United States; 4 Department of Biochemistry, University of Texas Southwestern Medical Center, Dallas, TX 75390, United States; 5 Department of Immunology, University of Texas Southwestern Medical Center, 5959 Harry Hines Boulevard, Dallas, Texas 75390, United States; 6 Center for the Genetics of Host Defense, University of Texas Southwestern Medical Center, 5959 Harry Hines Boulevard, Dallas, Texas 75390, United States; 7 Simmons Cancer Center, University of Texas Southwestern Medical Center, 5959 Harry Hines Boulevard, Dallas, Texas 75390, United States

## Abstract

Maintenance of intestinal epithelial integrity is essential for host defence, yet how epithelial junctional scaffolds connect to antimicrobial autophagy remains unclear. Here we show that distinct isoforms of the guanine nucleotide exchange factor GEF-H1 (mouse Arhgef2-207; human ARHGEF2–219) localize to adherens junctions in polarized intestinal epithelial cells through interaction with the adhesion molecule Nectin-3 and the cytoskeletal scaffold Afadin. Conditional deletion of Arhgef2-207 in the intestinal epithelium induces compensatory expression of the shorter Arhgef2-201, leading to loss of barrier integrity, activation of autophagy, and small-intestinal inflammation. In human intestinal organoids, infection with *Listeria monocytogenes* selectively targets the junction-associated ARHGEF2–219 isoform, triggering an isoform switch to ARHGEF2-201 and induction of autophagy through interaction with STING and LC3. This transition coincides with a loss of Na^+^/K^+^-ATPase and epithelial polarity. Together, these findings identify a pathogen-induced ARHGEF2 isoform switch that links junctional perturbation to autophagy and mucosal immune activation, defining a previously unrecognized pathway by which epithelial cells couple barrier disruption to cell-intrinsic host defence.

## Introduction

Disruption of intestinal epithelial integrity and impaired microbial sensing are hallmarks of chronic inflammatory bowel diseases (IBD) such as Crohn’s disease and ulcerative colitis ^1,2^. Autophagy plays a pivotal role in maintaining epithelial barrier function during infection by modulating inflammation, antimicrobial defense, and cell survival ^3,4^. Variants in multiple autophagy-related genes confer susceptibility to IBD, underscoring its importance in mucosal homeostasis ^5^. Autophagy encompasses catabolic processes that direct cytoplasmic material to lysosomes for degradation, while xenophagy, the selective autophagic clearance of invading microbes, constitutes a central epithelial defense mechanism ^6,7,8^.

The guanine nucleotide exchange factor GEF-H1 (ARHGEF2) links microtubules, actin filaments, and intercellular junction ^9–12^, but its epithelial functions remain incompletely defined. Originally described as a Dbl-family oncoprotein that activates RhoA, GEF-H1 has since emerged as an innate immune regulator, coordinating macrophage responses to pathogen-associated molecular patterns (PAMPs) such as double-stranded RNA and bacterial peptidoglycans ^13–16^. The GEF-H1–IKKε–IRF5 signaling axis is essential for defense against intracellular pathogens including *Listeria monocytogenes* ^16,17^.

*L. monocytogene*s, a Gram-positive facultative intracellular bacterium, targets epithelial junctions and cytoskeletal networks through a repertoire of internalins that mediate adhesion, invasion, and immune evasion. Entry into epithelial cells is driven by InlA binding to human E-cadherin and InlB interaction with the MET recepto*r* ^18–24^, whereas InlC and InlF remodel the actin cytoskeleton by engaging DNMBP and vimentin, respectively. Autophagy modulation by the bacterial proteins ActA and InlK further enables cytosolic survival ^19^. While phagocytosed *L. monocytogenes* triggers noncanonical autophagy, it can evade xenophagic clearance ^25,26^. However, the mechanisms by which *L. monocytogenes* induces autophagy and innate defenses in intestinal epithelial cells (IECs) remain poorly defined.

The *ARHGEF2* locus produces multiple GEF-H1 transcripts with distinct first exons, reflecting alternative promoter usage that may underlie cell type–specific expression and function. To define their roles in epithelial integrity and mucosal immunity, we generated isoform-specific knockout mice and developed antibodies that discriminate between long and short GEF-H1 mouse and human isoforms. These studies identify *ARHGEF2* as essential for intestinal homeostasis, encoding discrete isoforms that maintain epithelial barrier function and couple junctional disruption to autophagy-mediated defense against enteric pathogens.

## Results

### Distinct long ARHGEF2 isoforms are expressed in polarized intestinal epithelia.

**Figure 1 |:**
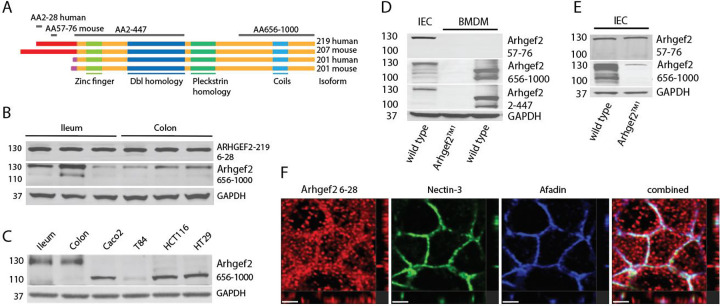
Human and mouse epithelial cells express long ARHGEF2 isoforms. **(A)** Domain alignment of human and mouse *Arhgef2* isoforms highlighting the unique N-terminal leader sequences of the long (red) and short (pink) variants. Antibody recognition regions used in this study are shown in grey, with corresponding amino acid positions indicated. **(B)** Immunoblot analysis of 3 independent derived ileal and colonic organoids confirming expression of the long ARHGEF2–219 isoform detected by an isoform-specific antibody. **(C)** Western blot analysis comparing ARHGEF2 expression in human intestinal organoids and colon cancer–derived cell lines. **(D)** Immunoblot analysis of ARHGEF2 isoform expression in mouse intestinal organoids and bone marrow–derived macrophages (BMDMs) from wild-type or *Arhgef2*^TM1^ mice. **(E)** Immunoblot analysis of ARHGEF2 isoforms in organoids derived from wild-type or *Arhgef2*^TM1^ mice. Protein sizes in all Western blots indicated in kDa. **(F)** Confocal microscopy showing ARHGEF2–219 localization at Nectin–Afadin junctions in human colonic epithelial cells (scale bars indicate 2μm).

We developed isoform-specific antibodies to distinguish human and mouse ARHGEF-2 isoforms that have unique N-termini that proceed the shared Dbl and Pleckstrin homology domains in shorter ARHGEF2 isoforms. (Fig. 1A). Human small and large intestinal epithelial organoids express the isoform ARHGEF2–219 (Fig. 1B), ARHGEF2–219 encodes a unique N-terminal segment of 164 amino acids, which shares 56% identity with the 220 amino acid N-terminus of murine *Arhgef2-207* (Fig.1A, C). The long isoform was also detected by antibodies raised against the common C-terminus confirming that ARHGEF2–219 and ARHGEF2-201 share downstream functional domains. Importantly, ARHGEF2–219 expression was absent in human colorectal adenocarcinoma-derived cell lines Caco2, T-84, HCT116 and HT-29 that expressed the shorter ARHGEF2-201 isoform instead (Fig 1C).

Arhgef2-207 -the mouse homologue of ARHGEF2–219- was the predominant isoform expressed in IECs isolated from wild-type mice which was confirmed by immunoblot and mass-spectrometry analysis following immunoprecipitation with isoform-specific antibodies (Fig. 1D; Tab. S1). In contrast, mouse bone marrow-derived macrophages (BMDMs) expressed only shorter GEF-H1 isoforms, lacking Arhgef2-207 (Fig. 1D; Tab. S2). Antibodies directed against AA 2–447 or AA 656–1000 of Arhgef2-201 also detected Arhgef2-207 confirming that Arhgef2-207 shared the same C-terminus (Fig. 1C, D). To further confirm isoform specificity, we examined *Arhgef2*^*TM1*^ mice, which contain a gene-trap insertion downstream of exons encoding the Arhgef2-201-specific N-terminus (IST13976A8) ^15^. These mice maintained Arhgef2-207 expression in IECs but lacked shorter GEF-H1 isoforms in both IECs and macrophages (Fig. 1D), confirming the non-overlapping usage of N-terminal exons between isoforms. ARHGEF2–219 was localized enriched at the level of Nectin-3-Afadin junctions in human colon epithelium (Fig.1F). ARHGEF2–219 was detected in and adjacent to Nectin-Afadin complexes and in puncta throughout the cytoplasm of IECs (Fig.1F). Collectively, these findings reveal that differentiated intestinal epithelial cells express long ARHGEF2 isoforms that are lost in intestinal cancer cell lines, which instead express only the shorter ARHGEF2 variants.

### Loss of epithelial Arhgef2-207 compromises barrier integrity and causes small-intestinal inflammation.

**Figure 2 |:**
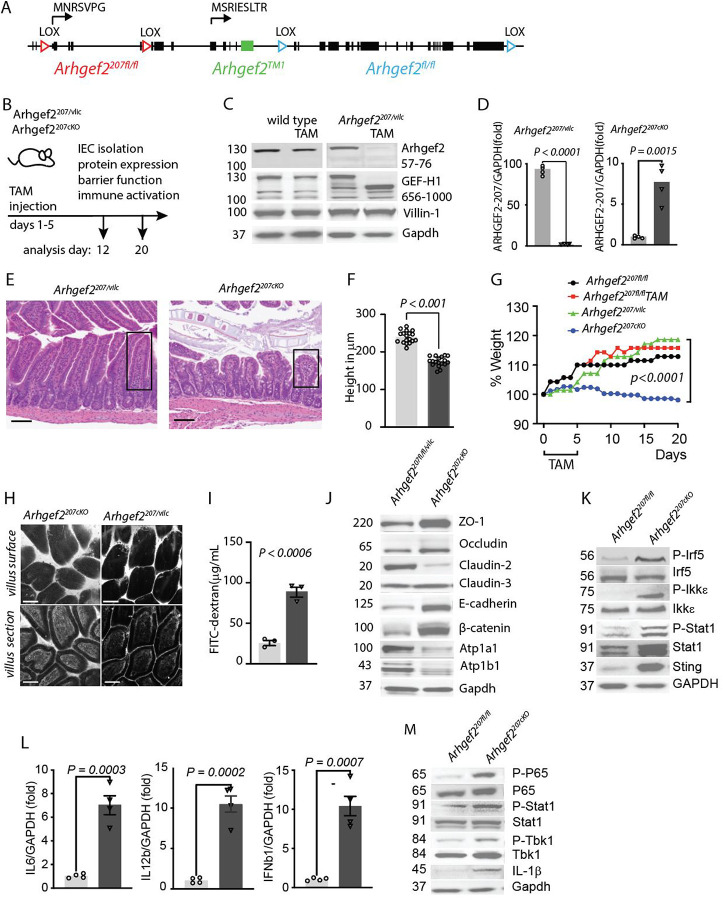
Inducible deletion of ARHGEF2-207 disrupts epithelial barrier integrity and elicits small-intestinal inflammation. **(A)** Targeting strategy and *loxP* integration sites in the *Arhgef2* gene enabling specific deletion of *Arhgef2-207* (red) or all *Arhgef2* isoforms (blue). The genetrap insertion site in *Arhgef2*^TM1^ mice is shown in green. Transcriptional start sites and N-terminal amino-acid sequences of *Arhgef2-219* and *Arhgef2-201* isoforms are indicated by arrows. **(B)** Experimental design for assessing *Arhgef2-207* function in intestinal epithelial cells (IECs). Mice received tamoxifen (TAM) for five days, and small-intestinal IECs were isolated for analysis of barrier function and inflammation at 7 and 15 days post-induction. **(C)** Immunoblot analysis of *Arhgef2* expression in IECs isolated from wild-type and *Arhgef2*^*207Vilc*^ mice before and after Cre-mediated deletion. **(D)** qPCR analysis of *Arhgef2-207* and *Arhgef2-201* mRNA expression before (grey bars) and after deletion of *Arhgef2-207* (black bars; n=4 per group). **(E)** Representative H&E-stained sections of small intestine 7 days after the end of Cre induction. Black frames mark villus height measured above crypts. *n* = 5 per genotype and time point. Scale bars, 100 μm. **(F)** Quantification of ileal villus height before (grey bars) and after Cre induction (dark grey bars); *n* = 20 per condition. **(G)** Body-weight development following 5 days of Cre induction in the indicated mouse strains (*n* = 5–7 per strain). **(H)** Confocal microscopy of ileal villi 1h after oral administration of FITC-dextran. **(I)** Quantification of FITC-dextran translocation into the bloodstream before (light grey bars) and after Cre induction (dark grey bars); *n* = 3 per condition. **(J)** Immunoblot analysis of apical and basolateral junctional proteins in isolated ileal IECs before and after Cre induction. **(K)** Western blot analysis of inflammatory transcription factors, innate-immune kinases and STING in ileal IECs before and after Cre induction. **(L)** qPCR analysis of inflammatory cytokine transcripts in the small intestinal lamina propria before (light grey bars) and after Cre induction (dark grey bars); n=4 per group. **(M)** qPCR analysis of transcription factors and immune regulators in mesenteric lymph nodes before and after Cre induction. Immunoblots and qPCR represent 3–5 repeated experiments. Data are presented as mean ± SEM with indicated *P* values analyzed by one-way ANOVA. All experiments were repeated twice and yielded consistent results.

To determine the functional relevance of distinct Arhgef2 isoforms, we generated conditional knockout mice targeting either Arhgef2-207 (*Arhgef2*^*207fl/fl*^) or all GEF-H1 isoforms (*Arhgef2*^*fl/fl*^) by flanking isoform-specific and shared exons with loxP sites (Fig. 2A). These mice were crossed with *Vil1-CreERT2* animals to achieve tamoxifen (TAM)-inducible, IEC-specific deletion (designated *Arhgef2*^*207vilc*^ and *Arhgef2*^*vilc*^, respectively; hereafter referred to as *Arhgef2*^*207cKO*^ and *Arhgef2*^*cKO*^ after TAM treatment).

Following five consecutive days of TAM administration, we analyzed small intestines and isolated IECs between 7 and 15 days post-induction (Fig. 2B). Deletion of Arhgef2-207 in *Arhgef2*^*207cKO*^ IECs led to compensatory upregulation of Arhgef2-201 protein, while TAM had no effect on GEF-H1 expression in wild-type controls (Fig. 2C). qPCR analysis confirmed increased Arhgef2-201 transcript levels and lack of Arhgef2-207 mRNA expression in IECs lacking *Arhgef2-207* (Fig. 1D).

Histological analysis of *Arhgef2*^*207cKO*^ mice revealed marked villus atrophy, epithelial disorganization, and a significant reduction in villus height 12 days post-TAM (Fig. 2E, F). Unlike control mice, *Arhgef2*^*207cKO*^ mice failed to gain weight during the 20-day observation period, consistent with impaired nutrient absorption and mucosal integrity (Fig. 2G).

To assess epithelial barrier function, we administered oral FITC–dextran (40 kDa) and analyzed its localization by confocal microscopy four hours later. In *Arhgef2*^*207cKO*^ mice, FITC–dextran penetrated the intercellular spaces and lamina propria of the small intestine, whereas *Arhgef2*^*207vilc*^ controls exhibited intact epithelial architecture with minimal FITC signal beyond the apical surface (Fig. 2H). Quantification of serum FITC–dextran confirmed a significant increase in systemic uptake in *Arhgef2*^*207cKO*^ mice (Fig. 2I), indicating compromised barrier function (Fig. 2I).

To assess the functional consequences of Arhgef2-207 loss, we analyzed junctional protein expression in IECs. Western blotting revealed elevated levels of ZO-1, Occludin, β-Catenin, and E-Cadherin in *Arhgef2*^*207cKO*^ IECs, while expression of Claudin-2 but not Claudin-3 was reduced (Fig.2J).

Deletion of Arhgef2-207 further caused the loss of the Na^+^/K^+^-ATPase subunits ATP1A1 and ATP1B1 that are required for the development of epithelial polarity ^27^.

At the molecular level, loss of Arhgef2-207 resulted in activation of host defense signaling in IECs, with increased phosphorylation of IRF5, IKKε, STAT1 and upregulation of STING compared to IECs isolated from *Arhgef2*^*fl/fl*^ controls (Fig. 2K). Correspondingly, the lamina propria of *Arhgef2*^*207cKO*^ mice showed elevated expression of *Ifnb*, *Il12b*, and *Il6* transcripts (Fig. 2L). Immune activation extended to mesenteric lymph nodes, where we observed increased phosphorylation of NF-κB p65, STAT1, and TBK1, as well as elevated IL-1β expression (Fig. 2M).

Together, these findings demonstrate that Arhgef2-207 is essential for maintaining the integrity of the intestinal epithelial barrier. Genetic ablation of *Arhgef2-207* induces compensatory upregulation of *Arhgef2-201*, which is insufficient to restore barrier integrity, leading to spontaneous small intestinal inflammation and heightened susceptibility to colitis. These results reveal non-redundant roles for GEF-H1 isoforms in the maintenance of intestinal epithelial homeostasis.

### Arhgef2-207 and Arhgef2-201 engage distinct junctional networks in polarized epithelia.

The impaired barrier function observed in *Arhgef2*^*207cKO*^ mice suggested a specific role for Arhgef2-207 in the establishment of epithelial polarity and apical junctional complexes. To define its spatial localization, we performed super-resolution imaging using Stimulated Emission Depletion (STED) microscopy, which offers lateral resolution of 40–60 nm, enabling distinction between tight junction (TJ) and adherens junction (AJ) components - resolution not achievable by conventional confocal microscopy.

**Figure 3 |:**
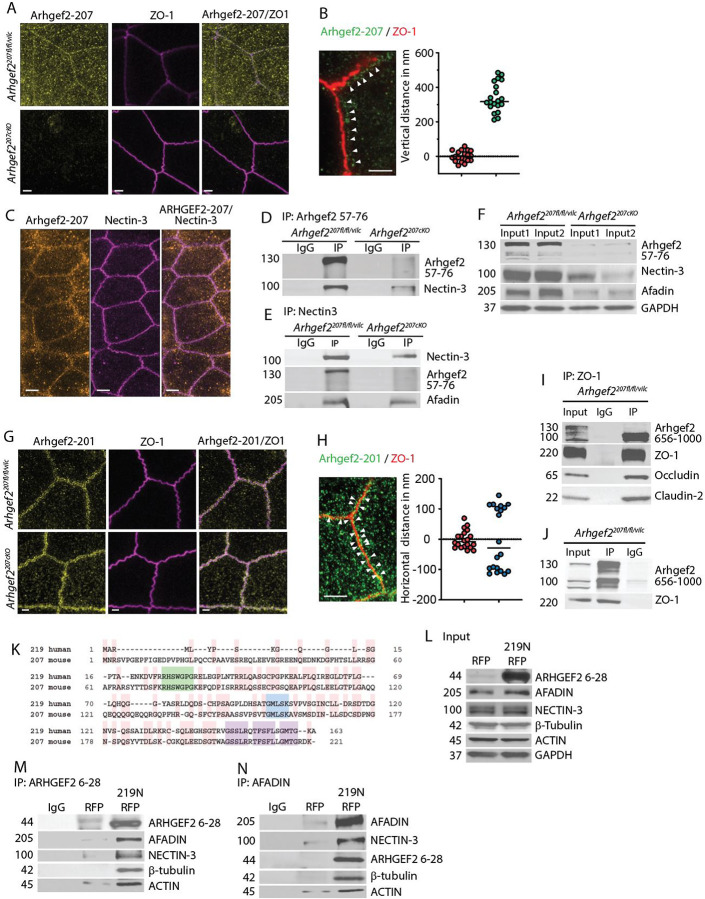
Isoform-specific coupling of Arhgef2 isoforms to epithelial junctional complexes. **(A)** Apical STED images of IECs in the mouse small intestine showing Arhgef2 localization at apical junctions. Scale bars, 1 μm. **(B)** Lateral STED view of junctional regions in mouse IECs with quantification of the distance from the midline of the ZO-1 signal. Arrows indicate puncta labeled by antibodies raised against amino acids 57–76 of Arhgef2-207. Scale bars, 1 μm. **(C)** Apical STED images of small-intestinal IECs stained with the indicated antibodies. Scale bars, 2 μm. **(D)** Immunoprecipitation of Arhgef2-207 before and after Cre induction in isolated IECs from mouse small intestine. **(E)** Immunoprecipitation of Nectin-3 before and after Cre induction in IECs from mouse small intestine. **(F)** Immunoblot analysis of lysates corresponding to the immunoprecipitation samples in (D, E). **(G)** Apical STED images of small-intestinal IECs showing junctional localization of Arhgef2-201. Scale bars, 1 μm. **(H)** Lateral STED images and quantification of ZO-1 distance from the epithelial midline. Arrows indicate puncta labeled by antibodies raised against amino acids 656–1000 of Arhgef2-201. Scale bars, 1 μm. **(I)** Representative immunoprecipitation of ZO-1 in isolated mouse IECs. **(J)** Input controls corresponding to the samples used in **(I)**. **(K)**Sequence alignment of the N-termini of mouse Arhgef2-207 and human ARHGEF2–219. Conserved residues are highlighted in pink; the conserved RHSWGPG (loop) motif in green; the GMLSK (helix seed) motif in blue; and the GSSLR–TFSF–GMTG (coiled-coil–like) region in grey. **(L)** Immunoblot analysis of human epithelial ARPE-19 cells expressing RFP or the RFP-tagged N-terminus of ARHGEF2–219, serving as input for panels **(M, N)**. **(M)** Immunoprecipitation of the RFP-tagged N terminus of ARHGEF2–219 and its associated complexes in ARPE-19 cells. **(N)** Immunoprecipitation of AFADIN and its associated complexes in ARPE-19 cells. All experiments were at least repeated twice and yielded consistent results.

In wild-type IECs, Arhgef2-207 localized to apical cell–cell contacts, specifically beneath the tight junction marker ZO-1 (Fig. 3A). Quantitative analysis revealed that Arhgef2-207 was positioned 340.0 ± 18.7 nm basally relative to the ZO-1 signal, consistent with localization to the adherens junction (Fig. 3B). Additionally, Arhgef2-207 was distributed in punctae across the apical cytoplasm, reflecting possible association with the apical F-actin and microtubule network (Fig. 3A, B). Staining specificity was confirmed by the absence of signal in *Arhgef2*^*207cKO*^ IECs using antibodies raised against amino acids 57–76 of the isoform-specific N-terminus (Fig. 3A).

The AJ consists of two concentric rings: an outer ring containing Nectin-Afadin complexes and an inner ring composed of E-cadherin–catenin assemblies ^29^. STED imaging demonstrated that Arhgef2-207 co-localized with Nectin-3 at the apical adherens junctions (Fig. 3C). Co-immunoprecipitation experiments from primary IECs confirmed complexes that contained Arhgef2-207, Nectin-3 and Afadin, detectable in *Arhgef2*^*207vilc*^ but absent in *Arhgef2*^*207cKO*^ IECs (Fig. 3D–F).

In contrast, the Arhgef2-201 isoform, detected using antibodies against a shared C-terminal epitope, enriched in a punctate distribution at the level of ZO-1, situated 96.5 ± 6.5 nm horizontally from the tight junction marker in both *Arhgef2*^*207vilc*^ and *Arhgef2*^*207cKO*^ mice (Fig3. G,H). Upon deletion of Arhgef2-207, Arhgef2-201 remained localized to TJs (Fig. 3G). Immunoprecipitation with antibodies directed against ZO1 pulled down the 100 kDa Arhgef2-201 isoforms but not the ARHGEf2-207 isoform (Fig. 3I). Interaction between ZO-1 and short GEF-H1 isoforms also occurred in the absence of ARHGEf2-207 in *Arhgef2*^*207cKO*^ IECs (Fig. 3J).

The N-terminal region shared by *ARHGEF2*-207 and *ARHGEF2*-219 contains a conserved triad of structural motifs—a short RHSWGPG loop, a proximal GMLSK helix-initiating element, and a downstream GSSLR–TFSF–GMTG coiled-coil–like cassette—that together resemble cytoskeletal interaction modules found in junctional scaffolds such as Afadin ^28–30^ (Fig. 3K). To examine whether this region mediates junctional or cytoskeletal associations, RFP fusion constructs encoding the N-terminus of ARHGEF2–219 were expressed in the non-transformed human epithelial cell line ARPE-19 (Fig. 3N). Immunoprecipitation using antibodies directed against amino acids 6–28 of ARHGEF2–219 identified the junctional scaffold Afadin, the adhesion receptor Nectin-3, and the cytoskeletal components β-tubulin and actin as interactors (Fig. 3M). Reciprocal immunoprecipitation for Afadin confirmed the presence of complexes containing the ARHGEF2–219 N-terminal domain together with Nectin-3, β-tubulin, and actin (Fig. 3N).

Together, these data reveal isoform-specific compartmentalization of *Arhgef2* in polarized epithelial cells. *ARHGEF2*-219 localizes to AJs, where it associates with Nectin-Afadin complexes and connects them to the actin–microtubule cytoskeleton, whereas *Arhgef2*-201 is enriched at tight junctions (TJs) in association with ZO-1. These findings demonstrate that distinct *Arhgef2* isoforms engage different junctional assemblies essential for epithelial barrier integrity, with the conserved N-terminal cassette of ARHGEF2 functioning as a structural interface between Afadin-Nectin complexes and the cytoskeletal network.

### Loss of Arhgef2-207 triggers autophagy in the intestinal epithelium.

**Figure. 4 |:**
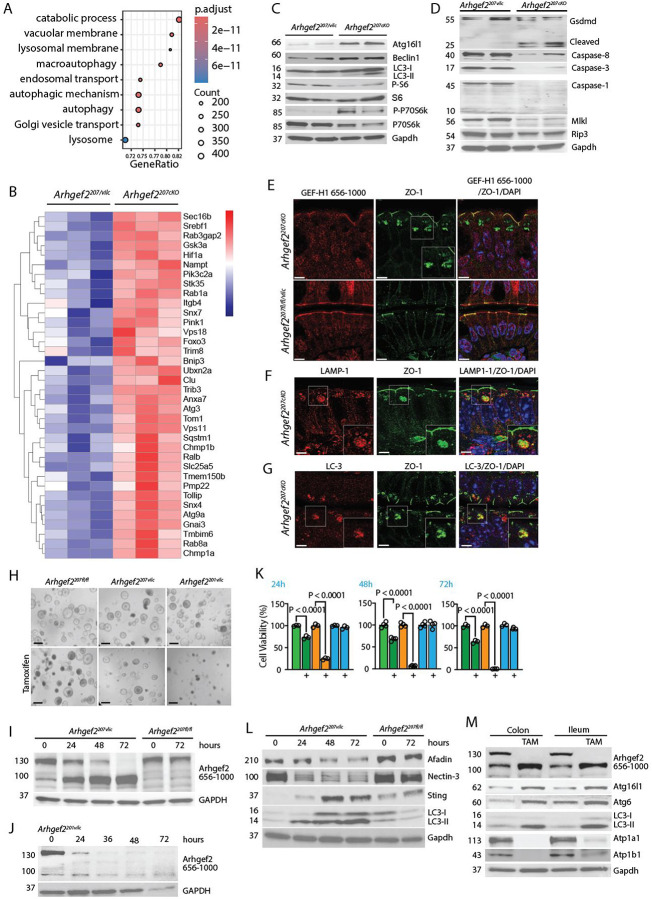
Loss of epithelial Arhgef2-207 drives an autophagy-dominated stress program compromising epithelial polarity. **(A)** Gene set enrichment analysis (GSEA) of differentially expressed genes in IECs following *Arhgef2-207* deletion in the small intestine. The top nine enriched signaling pathways are shown. **(B)** Hierarchical cluster analysis of autophagy-related genes upregulated upon *Arhgef2-207* deletion in IECs (three independent biological replicates). **(C)** Immunoblot analysis of autophagy-related proteins in IECs before and after Cre induction and *Arhgef2-207* deletion (two independent experiments are shown). **(D)** Immunoblot analysis of necroptosis- and autophagy-associated proteins in isolated IECs before and after Cre induction (two independent samples are shown). **(E–G)** Confocal microscopy of the small-intestinal epithelium before and after Cre-induced *Arhgef2-207* deletion. Larger square inserts represent magnification of cell compartments indicated by small squares. Scale bars, 5 μm. **(H)** Representative images of small-intestinal organoids from the indicated mouse strains before and after tamoxifen (TAM) treatment. Scale bars, 200 μm. **(I)** Immunoblot analysis of *Arhgef2* isoform expression in *Arhgef2*^*207Vilc*^ and *Arhgef2-207*^*fl/fl*^ organoids during TAM treatment. **(J)** Immunoblot analysis of *Arhgef2* isoform expression in *Arhgef2*^*201Vilc*^ organoids during TAM treatment. **(K)** Quantification of cell survival before and after TAM treatment in organoids derived from *Arhgef2207*^*Vilc*^ (green bars), *Arhgef2*^*201Vilc*^ (orange bars), and *Arhgef2-207*^*fl/fl*^ (blue bars ) mice. **(L)** Immunoblot analysis of junctional and autophagy-related proteins in *Arhgef2*^*207Vil-CreERT2*^ and *Arhgef2*^*201Vil-CreERT2*^ organoids after TAM treatment. **(M)** Immunoblot analysis of autophagy-related proteins in colon and small intestinal organoids derived from *Arhgef2*^*207Vilc*^ *mice* after TAM treatment.

To delineate the transcriptional consequences of ARHGEf2-207 loss, we performed RNA sequencing (RNA-seq) on IECs isolated from *Arhgef2*^*207vic*^ and *Arhgef2*^*207cKO*^ mice. Differential gene expression analysis identified 1,175 genes significantly regulated in *Arhgef2*^*207cKO*^ IECs (Table S3). Gene ontology enrichment revealed a marked upregulation of pathways associated with proteasomal degradation, lysosomal membrane organization, and autophagy (Fig. 4A). Consistent with this enrichment, *Arhgef2*^*207cKO*^ IECs exhibited increased expression of core autophagy genes, including *Foxo3*, *Atg3*, *Atg9a*, and *Hif1a* (Fig. 4B).

Immunoblotting confirmed upregulation of canonical autophagy proteins Atg16L1, Beclin-1, LC3-II and STING in targeted IECs (Fig. 4C). Phosphorylation of ribosomal protein S6 was decreased, alongside increased phosphorylation of S6 kinase (S6K), consistent with mTOR pathway suppression and induction of autophagic flux (Fig. 4C).

To assess whether epithelial stress responses extended to cell death pathways, we profiled markers of apoptosis, necroptosis, and pyroptosis. Surprisingly, *Arhgef2*^*207cKO*^ IECs exhibited reduced expression of cleaved caspase-3, caspase-8, and caspase-1, as well as necroptotic effectors MLKL and RIPK3 (Fig. 4D). In contrast we observed an increased expression of cleaved Gasdermin D (GSDMD) (Fig. 4D) that may be involved in the exocytosis of mucin granules by goblet cells and nonlytic IL-1β release in IECs ^31,32^.

We next examined whether ARHGEf2-207 deficiency promoted the formation of autophagy-related structures. IECs from *Arhgef2*^*207cKO*^ mice formed enlarged intracellular compartments enriched for the tight junction protein ZO-1, which were absent in *Arhgef2*^*207vilc*^ controls (Fig. 4E). These compartments colocalized with LAMP-1 and LC3, consistent with their identity as autophagosomes (Fig. 4F, G).

To determine whether autophagy was a direct epithelial response to ARHGEf2-207 loss, independent of immune or microbial cues, we generated intestinal organoids from *Arhgef2*^*fl/fl*^, *Arhgef2*^*207vilc*^, and *Arhgef2*^*201vilc*^ mice (Fig. 4H). In *Arhgef2*^*207vilc*^ organoids, tamoxifen-induced Cre activity led to progressive depletion of ARHGEf2-207 (130 kDa) over 72 hours, accompanied by upregulation of the ARHGEf2-201 isoform (110 kDa), replicating the *in vivo* switch observed in primary *Arhgef2*^*207cKO*^ IECs (Fig. 4I). Tamoxifen treatment had no effect on ARHGEF2 isoform expression in *Arhgef2*^*fl/fl*^ controls (Fig. 4I). In contrast, *Arhgef2*^*201vilc*^ organoids, which delete all ARHGEF2 isoforms, showed complete loss of GEF-H1 expression within 48 hours (Fig. 4J).

Functionally, ARHGEf2-207 deficiency reduced organoid viability by 20–35% (Fig. 4K), whereas complete loss of all GEF-H1 isoforms resulted in rapid and profound epithelial collapse (Fig. 4K). Thus, while ARHGEf2-201 may partially compensate for ARHGEf2-207 loss, it is insufficient to sustain long-term epithelial viability. Treatment of *Arhgef2-207*^*fl/f*^ organoids with TAM had no impact on cell viability (Fig. 4K).

Deletion of ARHGEf2-207 resulted in the downregulation of Afadin and Nectin-3, induction of STING and LC3 lipidation (Fig. 4L) in *Arhgef2*^*207vilc*^. TAM treatment of *Arhgef2*^*fl/fl*^ had no impact on junctional or autophagic protein expression (Fig 4L).

Importantly, ARHGEf2-207 deletion in both small and large intestinal organoids induced autophagy, mirroring the phenotype observed *in vivo*. This included upregulation of LC3-II, Atg16L1, and Beclin-1, as well as downregulation of Na^+^/K^+^-ATPase subunits ATP1A1 and ATP1B1 (Fig. 4M).

Together, these findings reveal that loss of Arhgef2-207 reprograms IECs toward an autophagy-dominant stress response with the upregulation of Arhgef2-201 and characterized by the accumulation of tight junction components within autophagosomes, transcriptional upregulation of autophagy effectors, and impaired polarity. This isoform-specific regulation of epithelial homeostasis underscores a non-redundant, protective role for ARHGEf2-207 in maintaining intestinal barrier integrity.

### Junctional ARHGEF2–219 is targeted by *L. monocytogenes* in the human epithelium.

The loss of epithelial barrier integrity and concurrent activation of autophagy and innate immune transcriptional programs upon *Arhgef2-207* deletion suggested that the long ARHGEF2 isoforms may serve in a key protective role in host defense against invasive pathogens. To test whether this mechanism is co-opted by enteric pathogens, we investigated the regulation of GEF-H1 isoforms during infection with *L. monocytogenes* which disrupt epithelial junctions during invasion and have evolved strategies to escape autophagic clearance ^7,8,23,24^.

**Figure 5 |:**
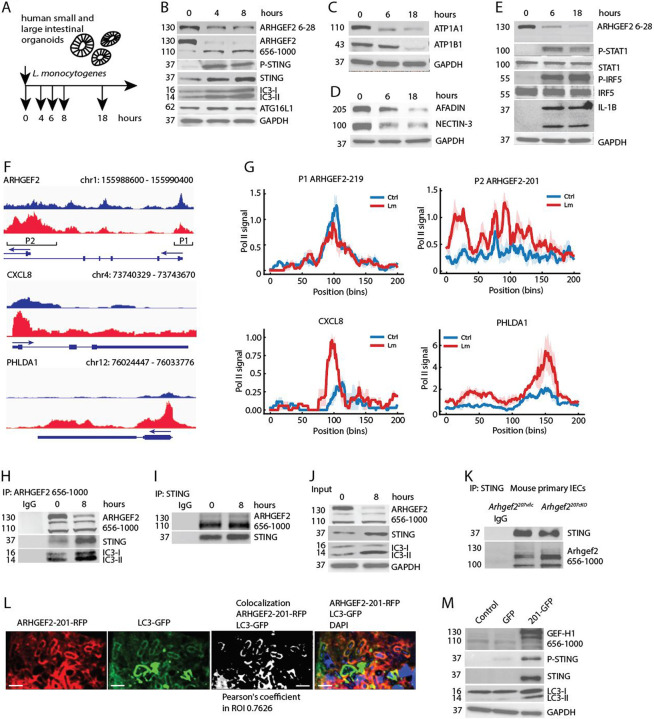
Listeria monocytogenes targets the long ARHGEF2 isoform while the short ARHGEF2 isoform induces autophagy in human epithelial cells. **(A)** Experimental design. Human small- and large-intestinal organoids were infected with *L. monocytogenes* to assess regulation of ARHGEF2 isoforms. **(B)** Immunoblot analysis of ARHGEF2 isoforms and autophagy-related proteins (LC3, ATG16L1, STING) in intestinal organoids before and after L. monocytogenes infection (8 h). **(C)** Immunoblot analysis of Na^+^/K^+^-ATPase subunits (ATP1A1, ATP1B1) in organoids before and after L. monocytogenes infection. **(D)** Immunoblot analysis of Afadin and Nectin-3 expression in organoids before and after L. monocytogenes infection. **(E)** Immunoblot analysis of innate immune signaling proteins in organoids before and after L. monocytogenes infection, including phosphorylated STAT1 and IRF5, and pro- and cleaved IL-1β. **(F)** Genome browser tracks showing Pol II CUT&Tag profiles from control (blue) and *Listeria monocytogenes*–infected (red) human intestinal organoids for 4 hours across the *ARHGEF2*, *CXCL8*, and *PHLDA1* loci (hg38). Two distinct ARHGEF2 promoters are evident: a proximal P1 promoter and an internal P2 promoter. Arrows indicate gene orientation and exons are shown relative to the PolII profile. **(G)** Promoter-centered Pol II CUT&Tag profiles (mean ± s.e.m.) for ARHGEF2 P1 (219-isoform TSS), ARHGEF2 P2 (201-isoform TSS), CXCL8, and PHLDA1, generated from ±1 kb windows centered on the Pol II peak summit (200 bins). Curves represent the mean of three biological replicates per condition (Ctrl, blue; Lm, red). **(H)** Immunoprecipitation of ARHGEF2 in human intestinal organoids before and after *L. monocytogenes* infection showing interaction with LC3 and STING complexes. **(I)** Reciprocal immunoprecipitation of STING detecting its association with ARHGEF2-201 but not ARHGEF2–219. **(J)** Quantification of ARHGEF2–STING complex formation after 8 h of *L. monocytogenes* infection. **(K)** Immunoprecipitation of STING from intestinal epithelial cells isolated from *Arhgef2*^*207Vlic*^ and *Arhgef2*^*207KO*^ mice. **(L)** Confocal microscopy of Caco-2 cells co-expressing RFP-ARHGEF2-201 and LC3-GFP during *L. monocytogenes* infection. Scale bars, 2 μm. **(M)** Immunoblot analysis of ARPE-19 cells expressing GFP or ARHGEF2-201–GFP for detection of STING, phosphorylated STING, and LC3-II. All experiments were repeated independently at least twice with consistent results except the CUT&Tag experiments which were carried out in triplicates.

Because *L. monocytogenes* invasion is mediated via internalin A binding to human E-cadherin ^23^, we employed human small and large intestinal organoids to model host-pathogen interactions (Fig. 5A).

Upon *L. monocytogenes* infection, ARHGEF2–219 protein levels declined sharply within 8 hours (Fig. 5B). This loss was accompanied by the induction of the short 110 kDa isoform, ARHGEf2-201, indicating an isoform switch in response to bacterial invasion (Fig. 5B). Strikingly, *L. monocytogenes* invasion led to the induction of an autophagic responses with increased expression of lipidated LC3, ATG16L1 and STING ^33^ (Fig. 5B). Listeria infection further led to the loss of Na^+^/K^+^-ATPase subunits ATP1A1 and ATP1B1 (Fig. 5C), and loss of Afadin and Nectin-3 (Fig. 5D), suggesting a disruption of junctional integrity and polarity upon cell invasion.

In parallel, human intestinal epithelial organoids activated canonical GEF-H1-dependent innate immune pathways, including phosphorylation of STAT1 and IRF5, and upregulation of IL1B expression, with subsequent IL-1β cleavage (Fig. 5E). These responses mirror GEF-H1-dependent immune activation previously characterized in macrophages ^16,17^.

Pol II CUT&Tag profiling revealed Listeria infection-specific changes in ARHGEF2 promoter usage (Fig. 5F). At the proximal ARHGEF2–219 (P1) promoter, Pol II occupancy showed only a modest reduction following 4 hours of *Listeria* infection (Ctrl: 0.289 ± 0.023, Lm: 0.245 ± 0.026 RPGC; fold-change 0.85, log_2_FC −0.24), and the difference did not reach statistical significance (Welch’s t-test, *p* = 0.28) (Fig. 5 F, G; Table S4). In contrast, the ARHGEF2-201 (P2) promoter exhibited a strong induction of Pol II occupancy after infection (Ctrl: 0.312 ± 0.055, Lm: 0.633 ± 0.050 RPGC; fold-change 2.03, log_2_FC 1.02; Welch’s t-test, *p* = 0.0127) (Fig..5F, G; Table S4).

We also quantified Pol II CUT&Tag signal at the CXCL8, and PHLDA1 promoters to confirm correct PoLII detection at genes induced in control and Listeria-infected human intestinal organoids (Fig. 5 F, G; Table S4). At the CXCL8 promoter, Pol II occupancy increased from 0.21 ± 0.03 to 0.80 ± 0.13 RPGC upon infection, corresponding to an approximately 3.9-fold induction (*p* = 0.036) (Fig 5E,F, Table S4). At the TSS of PHLDA1 Pol II signal rose from 1.19 ± 0.28 to 3.95 ± 0.75 RPGC, representing an approximately 3.3-fold increase (*p* = 0.053) (Fig.5. F, G; Table S4). Together, these data indicate that Listeria selectively enhances transcription initiation at the ARHGEF2 P2 promoter, consistent with infection dependent induction of the short ARHGEF2 isoform during rapid activation of inflammatory and stress-response transcriptional programs.

### ARHGEF2-201 interacts with STING and LC3 in *L. monocytogenes* induced autophagosomes

Immunoprecipitation of ARHGEF2 in human organoids before and after invasion with *L. monocytogenes* showed the interaction with autophagic protein complexes containing STING and LC3 that increased 8 hours post infection (Fig. 5G, J). The reverse immunoprecipitation with antibodies directed against STING revealed that ARHGEF2-201 but not ARHGEF2–219 interacted with STING (Fig. 5I, J). We also confirmed the interaction of Arhgef2-201 and STING in primary isolated IECs from Arhgef2^207vilc^ and Arhgef2^207KO^ mice where STING complexes showed increased association with Arhgef2-201 upon deletion of Arhgef2-207 and induction of autophagy (Fig 5K). Further, RFP tagged ARHGEF2-201 and LC3-GFP colocalized in *L. monocytogenes* containing autophagosomes in Caco-2 cells with a Pearson’s coefficient in the ROI of 0.76 (Fig. 5L).

Remarkably, the increased expression of ARHGEF2-201 by itself in IECs can induce autophagy. Expression of ARHGEF2-201-GFP but not GFP alone induced the expression of STING, STING phosphorylation and lipidated LC3-II in primary human epithelial cell line ARPE-19 (Fig.5M).

Collectively, these findings demonstrate that *L. monocytogenes* specifically targets the junction-associated isoform ARHGEF2–219 in Nectin-Afadin complexes in human IECs, leading to the upregulation of the ARHGEF2-201 isoform that interacts with STING and LC3. These interactions appear to govern autophagy and cell-autonomous immune responses in IECs. The disruption of ARHGEF2–219 function initiates a ARHGEF2-201 dependent autophagy-mediated host defense program, linking junctional targeting by *L. monocytogenes* to innate immune activation.

## Discussion

Our study uncovers a novel isoform-switching mechanism by which ARHGEF2 links epithelial polarity to autophagy and mucosal host defence. The long isoform, Arhgef2-207 in mice and its human orthologue ARHGEF2–219, localizes to apical AJs through Nectin-3 and Afadin, anchoring the long GEF-H1 isoform to the cytoskeleton of polarized intestinal epithelial cells. Expression of ARHGEF2–219 is restricted to differentiated epithelia and lost in colorectal cancer cell lines, which instead express the shorter ARHGEF2-201 isoform. Conditional deletion of *Arhgef2-207* in mice triggers compensatory expression of Arhgef2-201, which preserves cell viability but fails to restore polarity, leading to autophagy induction and intestinal inflammation. These findings identify ARHGEF2–219 as a non-redundant regulator of epithelial polarization and barrier integrity.

The conserved N-terminal module shared by ARHGEF2–219 and Arhgef2-207 forms a compact loop–helix–coiled-coil cassette resembling the actin-binding architecture of Afadin ^28–30^, suggesting a cytoskeletal anchoring function that couples junctional complexes to microtubule and actin networks. Mechanical or microbial disruption of this module likely liberates GEF-H1 from AJs, enabling the transcriptional induction and autophagic activity of the short isoform. Loss of Arhgef2-207 destabilizes junctions, reduces Na^+^/K^+^-ATPase expression, and drives the accumulation of tight-junction proteins within LC3- and LAMP1-positive autophagosomes. These features parallel the enhanced autophagy observed after pharmacological Na^+^/K^+^-ATPase inhibition and reveal how junctional perturbation can directly engage the autophagic machinery^34,35^.

Deletion or loss of Arhgef2-207 initiates a transcriptional program characterized by upregulation of STING and Atg16L1 and LC3 lipidation, whereas epithelial death pathways are restrained, with reduced caspase and necroptosis markers but enhanced Gasdermin-D processing - features consistent with non-lytic IL-1β release and mucosal repair ^31,32^. This epithelial-intrinsic autophagic response functions as a tiered defence system: quiescent at intact junctions yet rapidly mobilized upon their disruption.

During *L. monocytogenes* infection, the pathogen selectively targets ARHGEF2–219. Within hours of invasion, *L. monocytogenes* depletes ARHGEF2–219 and enhances ARHGEF2-201 expression, coinciding with loss of Na^+^/K^+^-ATPase. Promoter analysis demonstrates that Listeria infection did not significantly reduce ARHGEF2–219 promoter activation but enhanced activation of the specific promoter for the short ARHGEF2 isoforms. Active ARHGEF2 maybe be further released after cleavage of the N-terminus of ARHGEF2–219, as they share the same C-terminus, or release of ARHGEf2-201 from TJs ^10,36^. ARHGEF2-201 localizes to *L. monocytogenes*-containing autophagosomes, interacts with STING and lipidated LC3, and its overexpression alone is sufficient to induce STING phosphorylation and autophagic flux. This pathogen-triggered isoform switch thus couples epithelial junctional damage to innate immune signaling through the canonical GEF-H1–IKKε–IRF5 pathway ^16^, integrating xenophagy and inflammatory activation.

Key questions remain regarding the transcriptional and post-translational mechanisms governing ARHGEF2 isoform expression and degradation, the structural determinants mediating autophagic targeting, and the upstream cues that trigger isoform switching. Determining whether *L. monocytogenes* internalins or other microbial factors directly destabilize GEF-H1 junctional complexes will be essential to understanding how pathogens exploit this pathway.

From a translational perspective, ARHGEF2 isoform regulation may represent a critical determinant of epithelial resilience. Genome-wide association studies implicating autophagy genes, polarity factors, GEF-H1 and its interactor NOD2 in IBD susceptibility ^1–8,13,14,17,37^, suggest that defective maintenance or dysregulated switching of ARHGEF2 isoforms could shift the mucosa from adaptive repair to chronic inflammation. By linking epithelial polarity with autophagy and innate immunity, GEF-H1 acts as a molecular switch that coordinates homeostasis and defence at the mucosal surface. Modulating this pathway could restore barrier integrity while enhancing endogenous autophagic protection in inflammatory and infectious diseases of the intestine.

## Methods

Please see key resources list for antibodies, primers and chemicals used (Tab. S5)

### Mice

All experimental mice were sex-matched at 7–12 weeks of age using protocols approved by the Committee on Research Animal Care at the UT Southwestern Medical Center, Dallas under the protocol number 2020–102946. All animals were bred and housed in a pathogen-free animal facility according to the institutional guidelines. Up to 5 adult mice were housed in filtered cages with corn cob bedding and provided with standard chow and drinking water ad libitum. Cages were changed at least once a week in the laminar air-flow cabinet. Mice were subjected to 12 hours light: 12 hours dark cycles. *Arhgef2*^TM1^ mice were previously described ^15^. *Arhgef2*^*207fl/fl*^ mice were created with loxP site insertion before exon 1 and after exon 4 of Arhgef2-207 without affecting transcriptional start sites for other GEF-H1 isoforms. To eliminate all functional GEF-H1 isoforms we also inserted loxP sites upstream and downstream of exons that encode for the common domains of all GEF-H1 isoforms before exon 2 and after exon 22 of Arhgef2-201 creating *Arhgef2*^*fl/fl*^ mice. *Arhgef2*^*207fl/fl*^ or *Arhgef2*^*fl/f*^ *mice* were crossed with mice expressing tamoxifen (TAM)-inducible Cre recombinase under the control of the villin1 promoter, *Vil-CreERT2* mice (The Jackson Laboratory, #020282) *Arhgef2*^*207vilc*^ and *Arhgef2*^*207cKO*^ after TAM treatment). Both *Arhgef2*^*207cKO*^ and *Arhgef2*^*fl/fl*^ were treated with TAM for 5 consecutive days and small intestinal epithelial cells isolated and analyzed 7–15 days later. Gene targeting was carried out in the Transgenic Technology Center Core at UTSW. C57BL/6 wild type mice were from Jackson Laboratory (Bar Harbor, ME, USA). For inducible gene ablation, tamoxifen (MilliporeSigma, Cat# C8267) was dissolved in corn oil at a final concentration of 20 mg/mL and i.p. injected into mice at the dose of 75 mg/kg body weight for five consecutive days.

### Isolation of primary IECs

Approximately 5 cm of small intestinal tissue was dissected, inverted on plastic tubes and extensively washed in PBS. Intestines were placed in 2 mM EDTA in PBS for 30 min on the rotator at 4C. Tissue fragments were placed in 10 mL of 0.1% BCS and pipetted up and down. Pipetting and 0.1% BCS solution exchange was repeated 6 times. Fractions 4–6 were collected, and IECs were collected by centrifugation.

### Analysis of FITC-dextran in the intestine and serum

Mice were given 0.25 mg/g of Fluorescein isothiocyanate–dextran (FITC-dextran; average MW 4,000; Sigma-Aldrich Cat# 46944) solution in PBS by oral gavage. After 4 hours, mice were anesthetized and blood was collected by cardiac puncture and small intestines harvested for live tissue imaging by confocal microscopy. During 4 hours of experiment, mice were deprived of food and water. Presence of FITC-dextran in the serum was analyzed by spectrophotometric measurement on Multi-Mode Microplate Reader (Synergy HT) at 485/528 nm (excitation/emission). A Leica Stellaris confocal microscope was used for image acquisition.

### RNA sequencing and analysis

Total RNA was isolated from isolated IECs from wild type, using a RNeasy Micro kit (Qiagen). Libraries were synthesized using TruSeq Stranded mRNA sample preparation kit from 500 ng of purified total RNA and indexed adapters according to the manufacturer’s protocol (Illumina). The final libraries were quantified using a Qubit fluorometer (Agilent Technologies), and RT-qPCR was performed using the Kapa Biosystems library quantification kit according to the manufacturer’s protocol. Pooled libraries were subjected to 35 bp paired-end sequencing according to the manufacturer’s protocol (Illumina NextSeq 500). The targeted sequencing depth was set at 30 million paired-end reads per sample. Blc2fastq2 Conversion software (Illumina) was used to generate demultiplexed Fastq files. The adaptors were trimmed using Trim Galore (v0.6.4). The trimmed reads were aligned to mouse genome (mm10) using STAR (2.7.3a). Subsequently, the mapped reads were quantified using the featureCounts of the Subread (v1.6.3) package. Genes with low expression (genes with an expression value of zero in more than 30% of the samples) were removed before subsequent analysis. Gene expression was normalized using the Voom method in the R package “limma” (v 3.50.3). Differentially expressed genes were also identified using this software. Gene set enrichment analysis of differentially expressed genes was performed using the R package “clusterProfiler” (v4.2.2). SeqMonk Mapped Sequence Data Analyzed 1.48.1 was used for DEseq2 analysis and initial visualization. Heat maps were generated using R package pheatmap (v1.0.12).

### CUT&Tag analysis

CUT&Tag libraries for Pol II were generated from human intestinal organoids exposed to vehicle or *Listeria monocytogenes* (3 biological replicates). Nuclei were isolated from Listeria-infected and control human intestinal organoids by gentle douncing in NE1 buffer. CUT&Tag was performed on 100,000 nuclei per sample following the established protocol ^38^. Briefly, nuclei were immobilised on concanavalin A-coated magnetic beads and incubated overnight at 4°C with anti-RPB1 (Pol II) antibody (CST 2629) in digitonin-containing antibody buffer. After washing, a mouse secondary antibody and pre-loaded pAG-Tn5 adapter complex (EpiCypher) were sequentially applied, followed by tagmentation at 37°C for 1 h. DNA was released by heat in SDS buffer, neutralized, and purified by spin columns. Libraries were generated using NEBNext High-Fidelity PCR Master Mix and Nextera i5/i7 indexed primers, amplified for 11 cycles, size-selected with AMPure beads, quantified, assessed on a TapeStation, pooled equimolarly, and sequenced on the NovaSeq X Plus (PE150; ~20 million reads/sample). Reads were aligned to hg38, filtered to remove PCR duplicates and ENCODE blacklist regions, and converted into normalized genome-wide coverage tracks. Two ARHGEF2 promoters (P1 and P2) were defined from Pol II enrichment and CAGE TSS clusters and represented as ±1 kb windows around their Pol II summits. Pol II signal for each sample was quantified as the average normalized signal across all bins within each promoter window. Condition-specific means and variability were calculated, and differences between Ctrl and *Listeria* conditions were evaluated using Welch’s t-test. Promoter-level changes were visualized using averaged Pol II profiles and bar plots (mean ± s.e.m.) with associated log_2_ fold-changes and P values.

### Mouse small and large intestinal organoid culture

Mouse small intestinal and colon organoids were derived as previously described. ^39,40,41^. Small intestinal (15–20 cm) and colonic (3–6 cm) segments were harvested, flushed with cold PBS, opened longitudinally, and washed repeatedly until clear. Tissue was cut into ~2 mm pieces and washed 15–20 times in cold PBS to remove debris. Fragments were incubated in Gentle Cell Dissociation Reagent (StemCell Technologies, #100–0485) for 15 min (small intestine) or 20 min (colon) on a rocking platform at room temperature. Released crypts were filtered (70 μm), pelleted (290 × g, 5 min, 4 °C), and washed in PBS + 0.1% BSA. Crypt-enriched fractions were resuspended in DMEM/F-12 (15 mM HEPES) and mixed 1:1 with Matrigel^®^. Approximately 500 crypts were plated per well of a 24-well plate as domes and polymerized for 10 min at 37 °C. Cultures were maintained in complete epithelial organoid growth medium with medium changes twice weekly. For TAM-induced gene ablation, organoids were treated with 250 nM Z-4-Hydroxytamoxifen (Sigma-Aldrich, Cat# H7904) for 3 days, followed by medium exchange to Intesticult medium without Z-4-Hydroxytamoxifen.

### Human organoid culture.

Human small intestinal and colon tissue samples were collected in the Division of Digestive and Liver Diseases at the University of Texas Southwestern Medical Center under the IRB Protocol Number STU-112010–130,” Registry and Biorepository for the study of Gastrointestinal Inflammatory Diseases”. Epithelial organoids were established from endoscopic biopsies of the ascending colon obtained with informed consent from de-identified donors. Organoids were generated following a modified protocol based on ^42 43^. Briefly, biopsies were digested in 2 mg/ml collagenase IA, filtered through a 70 μm strainer, and pelleted (150g, 5 min). The epithelial fraction was resuspended in cold wash medium (DMEM/F12 50:50 with 10% FBS, 1× Pen/Strep) and embedded in 30 μl Matrigel. After polymerization (10 min, 37 °C, inverted), 450 μl of complete epithelial organoid medium was added, supplemented for the first 48 h with CHIR 99021 (2.5 μg ml^−1^) to activate Wnt/β-catenin signaling. ROCK inhibitor (Y-27632) and TGFBR1 inhibitor were included to prevent anoikis and suppress serum-derived TGF-β activity. Cultures were passaged 1:1–1:2 depending on organoid density and morphology. Images of organoid cultures were taken on day 5 with a Zeiss inverted microscope.

### Cell lines

Arpe-19 or Caco-2 cells were seeded on 8-well-chambered coverslip slides (ibidi GmbH, cat# 80806) at 2.5 × 10^4^ per well, and cells at 60–70% confluency were transfected the next day using Lipofectamine 3000 reagent (Invitrogen Cat# L3000015) according to the manufacturer’s protocol. Typically, 50–200 ng of total plasmid DNA was transfected into each well. All expression plasmids encoded for full-length proteins, either untagged or C-terminally tagged with Flag epitope or fluorescent proteins. On the day after transfection, the cells were either used for live cell imaging or fixed and stained for immunofluorescence microscopy.

### Real-time quantitative-PCR

Total RNA was isolated using the RNeasy Mini Plus kit (Qiagen, Cat# 4134). cDNA was prepared from RNA using a PrimeScript RT Reagent cDNA Synthesis Kit (Takara, Cat RR0373a). Real-time qPCR was performed using PowerTrack SYBR Green master mix (Applied Biosystems, Cat# A46109) with gene specific primers and and relative expressions were calculated using the ΔΔCT method ^44^. Gene expression was normalized to *GAPDH*. All experiments were repeated at least twice. The specific primer sequences are listed in Tab. S5.

### Immunostaining

Immunofluorescence staining of mouse and human intestinal tissue Formalin-fixed paraffin-embedded (FFPE) sections of mouse and human small intestine were baked (60 °C, 30 min), deparaffinized in xylene, and rehydrated through graded ethanols. Antigen retrieval was performed in Tris-EDTA buffer (10 mM Tris, 1 mM EDTA, pH 9.0) at 110 °C for 10 min. Sections were permeabilized with 0.25% Triton X-100 and blocked with 5% normal donkey serum. Slides were incubated overnight at 4 °C with primary antibodies against target proteins, followed by sequential incubation with fluorescent secondary antibodies (Alexa Fluor 488, 594, or 647; ThermoFisher) for confocal imaging, or Abberior STAR RED/ORANGE conjugates for STED microscopy. Secondary antibodies were diluted in blocking buffer and applied for 1 h at room temperature. After washing in 0.05% PBST and PBS, nuclei were counterstained with DAPI and mounted in Abberior Mount Solid Antifade medium. Colocalization studies were carried out in Imaris image analysis software after image acquisition using a Leica Stellaris confocal microscope or Abberior facilty line microscope.

### Western blots and immunoprecipitations

BMDMs were lysed in RIPA buffer (10 mM Tris-HCl, pH 8.0, 1 mM EDTA, 0.5 mM EGTA, 1% Triton X-100, 0.1% sodium deoxycholate, 0.1% SDS, 140 mM NaCl) supplemented with protease and phosphatase inhibitors (Thermo Fisher, #78430). Lysates were resolved by SDS–PAGE and transferred onto PVDF membranes. Membranes were blocked with 5% milk or BSA and incubated overnight at 4 °C with primary antibodies, followed by HRP-conjugated secondary antibodies (anti-rabbit IgG-HRP, #7074; anti-mouse IgG-HRP, #7076; Cell Signaling Technology, 1:3000). Detection was performed using enhanced chemiluminescence (Immobilon Western, Millipore, Cat#WBKLS010). Antibodies used included rabbit anti-Arhgef2-201 (aa 656–1000; Abcam, ab155785), sheep anti-mouse GEF-H1 (Exalpha, X1089S), and custom rabbit antibodies against Arhgef2-207 (aa 57–76, Pacific immunology corp. Ramona, CA, Cat#PAC17843/4) and human ARHGEF2–219 (aa 6–28, Pacific immunology corp. Ramona, CA, Cat#PAC17845/6). Additional antibodies are listed in Tab. S5. For immunoprecipitation, cells were lysed on ice for 20 min in IP lysis buffer (Thermo Fisher, #87787), cleared by centrifugation, and preincubated with protein G agarose (Thermo Fisher, #20423) for 1 h at 4 °C. Precleared lysates were incubated overnight at 4 °C with primary antibodies, followed by 4 h rotation with protein G agarose. Beads were washed three times with lysis buffer and eluted in 1× SDS sample buffer at 95 °C for 10 min before immunoblot analysis.

### Quantification and statistical analysis

Statistical analysis used GraphPad Prism 9 software (GraphPad Software, USA). Statistical parameters, methods, and significance are reported in the results section, within figure legends and in the method details. The data are presented as mean with standard error of the mean). The data were considered statistically significant when p<0.05. Image analysis was conducted in Imaris. All experiments were repeated independently at least twice with reproducible results, except RNA seq and CUT&Tag analysis, which were performed once in duplicate or triplicate samples.

## Supplementary Material

Supplementary Files

This is a list of supplementary files associated with this preprint. Click to download.

- SupplementaryTable3.xlsx
- SupplementaryTable1.xlsx
- SupplementaryTableS5.pdf
- SupplementaryTable2.xlsx
- SupplementaryInformation.pdf
- SupplementaryTable4.xlsx

## Data Availability

Cell lines generated in this study are available upon request to lead contact. Data and code availability. RNAseq and CUT&Tag data have been deposited at GEO: GSE311695. Accession numbers for individual datasets are listed in the key resources table. The accession numbers for published datasets analyzed in this study are listed in the key resources table. Original western blot images have been deposited at Mendeley and are publicly available as of the date of publication. The DOI is listed in the key resources table. This paper does not report original code. Any additional information required to reanalyze the data reported in this paper is available from the lead contact upon request.

## Data and code availability

https://www.ncbi.nlm.nih.gov/geo/query/acc.cgi?acc=GSE311695
